# Rapid and high efficiency transformation of *Chlamydomonas reinhardtii* by square-wave electroporation

**DOI:** 10.1042/BSR20181210

**Published:** 2019-01-11

**Authors:** Liang Wang, Lijing Yang, Xin Wen, Zhuoya Chen, Qiaoying Liang, Jialing Li, Wen Wang

**Affiliations:** 1 The Key Laboratory of Biotechnology for Medicinal Plant of Jiangsu Province, School of Life Sciences, Jiangsu Normal University, Xuzhou 221116, China; 2 College of Health Sciences, Jiangsu Normal University, Xuzhou 221116, China

**Keywords:** Chlamydomonas reinhardtii, electroporation, microalgae, square-wave, transformation

## Abstract

*Chlamydomonas reinhardtii*, the unicellular green algae, is the model organism for studies in various physiological processes and for bioindustrial applications. To explore the molecular mechanisms underlying physiological processes or to establish engineered cell lines, the exogenous DNA needs to be integrated into the genome for the insertional mutagenesis or transgene expression. However, the amount of selected marker DNA is not seriously considered in the existing electroporation methods for mutants library construction. Here, we reported a rapid-and-high-efficiency transformation technique for cell-walled strains using square-wave electroporation system. The final yield with this electroporation method was 2–6 × 10^3^ transformants per μg exogenous DNA for cell-walled strains in a strain-dependent manner. In general, this electroporation technique was the easy and applicable way to build a mutant library for screening phenotypes of interest.

## Introduction

*Chlamydomonas reinhardtii* (*C. reinhardtii*), a unicellular green alga, is an ideal model eukaryotic organism in many biological processes, including organelle biogenesis, biodiesel metabolism, photosynthesis, and cell cycle events [1–3]. *Chlamydomonas* cells are haploid during vegetative generation which makes it much easier for phenotypes analysis than higher plants and animals, especially the phenotypes caused by recessive mutations. Another advantage is that the whole genome of *Chlamydomonas* is available, making the genetic mapping possible [1,4]. Thus, forward genetic screen is the easy and effective approach to study the functions of new genes in *Chlamydomonas* cells.

For forward genetic analysis, mutagens and insertional fragments for mutagenesis are both the effective methods on *Chlamydomonas* cells. However, mutagens often make genetic mapping difficult for frequently bringing linked mutations and/or multiple sites mutations. Next-generation sequencing is the best way to solve this problem [5]. Several approaches are applied for mapping the insertional sites, including reverse PCR, plasmid rescue, thermal asymmetric interlaced PCR (TAIL-PCR), restriction enzyme site-directed amplification PCR (RESDA-PCR), SiteFinding PCR, hairpin-PCR, *Chlamydomonas* MmeI-based insertion site Sequencing (ChlaMmeSeq) [6–10], etc. Meanwhile, some strategies have been successfully applied to maintain the insertional mutant library for further analysis [10–12].

To integrate exogenous DNA fragment into the nuclear genome, various techniques have been developed for transformations of *Chlamydomonas* cells. The old and successful biolistic technology could deliver exogenous DNA into the genome [13]. However, this method needs a special DNA delivery system and yields few transformants. Glass beads transformation method is applied until now as it does not need special equipment and the procedure is relatively simple [14–17]. Although more transformants are obtained with this method, the primary defect is that cells should be cell-wall deficient or cell-wall removed before the glass beads agitation is applied. In addition, more and more new approaches are utilized for transformations of *Chlamydomonas* cells, such as transformations mediated by *Agrobacterium*, nanoparticles, microelectrode for DNA delivery [18–21]. Nevertheless, these techniques are either not widely employed or are with low transformation frequency.

The widely used transformation method is electroporation, which is the most effective technique for DNA incorporation [22,23]. It is reported that the transformation efficiency with this method is approximately 10^5^ transformants per μg exogenous DNA [23]. However, this method is based on the cell-wall-deficient strains or cell-wall-removal cells and leads as many as three copies of exogenous DNA fragments into the genome. Improved electroporation technique is applied for wild-type cells without cell-wall removal by the square electric pulse generating electroporator NEPA21, which yields approximately 0.4–3 × 10^3^ transformants per μg exogenous DNA with 400 ng DNA per trial [24]. However, the amount of antibiotic-resistance DNA fragments per trail is not seriously considered in the electroporation methods. Based our experiences on the study of electroporation with the decay-wave-pulse-type electroporator BTX ECM630 for *Chlamydomonas* cells, transformation efficiency for building the mutant library is 2–3 × 10^3^ transformants per μg DNA fragments [25,26].

To build a multipurpose insertional mutant library for forward genetic screens, the amount of insertional DNA fragments encoding selectable markers should be as less as possible, considering difficult identification of insertional sites by multiple insertional mutagenesis. Meanwhile, reducing the negative effect on cells with square-wave electroporation is the way to enhance higher transformation efficiency. In the present study, we present a square-wave electroporation method with as less as 100 ng (400 ng ml^−1^) antibiotic-resistance DNA fragments per cuvette that could highly yield transformants without cell-wall removal.

## Materials and methods

### Cell culture and strains

*C. reinhardtii* wild-type strain 21gr (CC-1690, wild-type, mt+), 6145C (CC-1691, wild-type, mt−), CC-125 (137c, wild-type, mt+), CC-124 (137c, wild-type, mt−) were originally provided from *Chlamydomonas* Resource Center, University of Minnesota, U.S.A., and kept in our laboratory. Cells were cultured in Tris-acetate-phosphate (TAP) plate at 23 ± 0.5°C under 14/10 h light/dark cycles with light intensity of 8000 Lx [25,27].

### Preparation of exogenous *aphVIII* DNA

The plasmid pJMG, carrying the *aphVIII* gene (paromomycin-resistance cassette) was originally modified from pSI103 (*Chlamydomonas* Resource Center, U.S.A., https://www.chlamycollection.org) and obtained from Dr. Junmin Pan’s laboratory [28]. The plasmids pJMG expanded in DH5α *E. coli* were purified with SanPrep Column Plasmid Mini-Preps Kit (Sangon Biotech, China). DNA fragments carrying the *aphVIII* gene were digested with restriction enzyme QuickCut *Eco*RI (Takara, Japan) and extracted by SanPrep Column DNA Gel Extraction Kit (Sangon Biotech, China). All DNA plasmids and fragments were quantified by NanoDrop 2000 (Thermo Scientific, U.S.A.).

### Square-wave electroporation

Cells were grown in liquid TAP medium under constant aeration and continuous light with light intensity of 8000 Lx until the cell density reached 1.0–2.0 × 10^7^ cells ml^−1^. Then, the cells stock medium was inoculated into fresh liquid TAP medium for the concentration of ∼1.0 × 10^6^ cells ml^−1^ and grown under continuous light for 18–20 h until the cell density was ∼4.0 × 10^6^ cells ml^−1^. Then cells were collected by centrifugation at 1250 ***g*** for 5 min at room temperature, washed and resuspended with pre-chilled TAP medium containing 60 mM sorbitol (Sigma, U.S.A.), and iced for 10 min. Then, 250 μl of cell suspension (corresponding to 5.0 × 10^7^ cells) were placed into pre-chilled 0.4 cm electroporation cuvette (BTX, U.S.A.) with 100 ng *aphVIII* DNA fragments [29]. Electroporation parameters of BTX ECM830 electroporation apparatus (BTX, U.S.A.) were indicated in the text or tables for different trials. Pulse interval time of 100 ms was constant for all trials. Voltage, pulse number, pulse length were evaluated for optimization of transformation conditions. For high transformation efficiency, the total time of the electroporation procedure was less than 1 h.

The cuvette was immediately placed on ice for 10 min after electroporation. Finally, the cell suspension was transferred into 50 ml conical centrifuge tube containing 10 ml TAP medium with 60 mM sorbitol for overnight recovery at dim light by slowly shaking. After overnight recovery, cells were recollected and plated with starch embedding method onto 1.5% (w/v) agar TAP plate with 10 μg ml^−1^ paromomycin (Sigma, U.S.A.). Plates were then incubated at 23 ± 0.5°C under continuous illumination with light intensity of 8000 Lx. Colonies of paromomycin-resistant transformants were visible and counted 5–7 days later. Photo Images for colonies were processed with Adobe Photoshop and Illustrator softwares (Creative Suite 6 edition, Adobe Systems Incorporated, U.S.A.). Graphs for transformant numbers were processed with GraphPad Prism 7 (GraphPad Software, U.S.A.)

### Colony PCR

To quickly identify the colonies containing the transformed *aphVIII* insert, *Chlamydomonas* colony PCR method was employed [30]. Briefly, cells suspended in 50 μl 5% (w/v) Chelex-100 (Bio-Rad, U.S.A.) were boiled for 10 min followed by immediately vortexed rigorously for 20 s. After on ice for 2 min, 1 μl of supernatant was prepared by centrifugation at 14000 rpm, 5 min for PCR template. The 264 bp DNA fragment was amplified by TransTaq HiFi DNA Polymerase (TransGen Biotech, China) with a forward primer F1 (5′-GATTCCCGTACCTCGTGTTG-3′) and a reverse primer R1 (5′-TCGTCCAGATCCTCCAAGTC-3′), using 29 cycles of thermal denaturation for 30 s at 97°C, annealing for 30 s at 58°C, and extension for 30 s at 72°C. PCR products were visualized and analyzed by running 1% (w/v) agarose gel and Gel Imaging Systems (Bio-Rad, U.S.A.).

## Results

### Optimization of square-wave electroporation method

From our previous study of electroporation with the decay-wave-pulse-type electroporator BTX ECM630, the applicable parameters for electroporation of *Chlamydomonas* cells is voltage of 800 V with electrical impedance of 1575 Ω, and capacitance of 50 μF [25]. The average of final pulse length is 10–14 ms for the successful electroporation trials. However, the pulse length of electroporation is uncontrollable and the number of transformants is unstable. To induce exogenous DNA into the cell, enough and controllable electric pulse time is important, on the other hand, low transformation efficiency will occur due to the long electric pulse time leading to the cells death or the short electric pulse time failing to incorporate the DNA into the cells.

**Figure 1:**
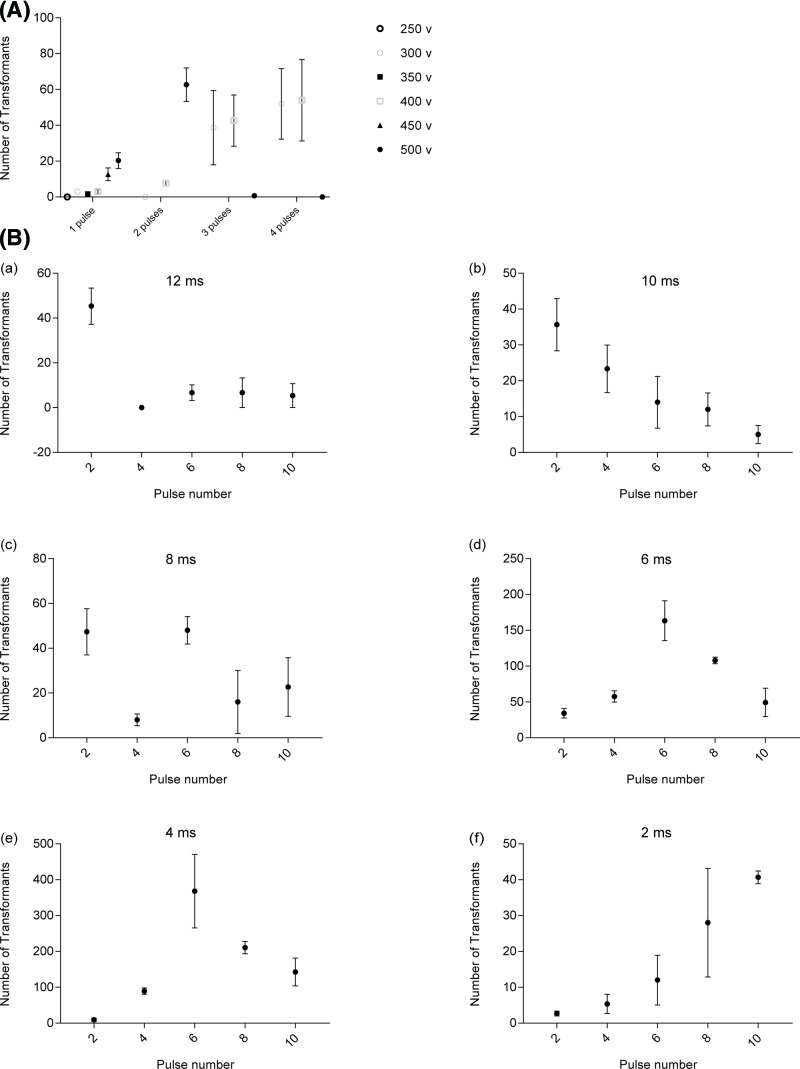
Optimization of transformation efficiency using square-wave electroporator *C. reinhardtii* strain 21gr cells were electroporated with 100 ng *aphVIII* DNA fragments and 250 μl cell suspension with the cell density of 2 × 10^8^ cells ml^−1^ in a 0.4 cm electroporation cuvette. (**A**) Effects of voltage and pulse number on transformation efficiency. The number of transformants was plotted at 250, 300, 350, 400, 450, 500 V with different pulse number as indicated. In all transformations pulse length and pulse interval were kept constant at 12 and 100 ms. (**B**) Effects of pulse length and pulse number on transformation efficiency at voltage of 500 V. The number of transformants was plotted at pulse numbers of 2, 4, 6, 8, 10 with different pulse length as indicated (a–f). The numbers of transformants were shown as mean ± SEM in three independent experiments.

To explore best characteristic parameters of square-wave-pulse-type electroporator BTX ECM830 for wild-type 21gr cells, one pulse number with 12 ms pulse length at different voltages were evaluated based on optimized pulse length from the decay-wave-pulse-type electroporator BTX ECM630. At low voltages (250 V, 300 V, 350 V, 400 V), there were almost no transformants (Figure 1A). The number of transformants was a bit increased at higher voltages (450, 500 V) (Figure 1A). Higher voltage of 800 V was evaluated too, however, the available range of pulse length was 10–600 μs at the high voltage mode (30–3000 V). At conditions of 800 V and 600 μs with one pulse, none of the transformants were obtained (data not shown). Probably electroporation time was not long enough for DNA delivery into the cell, based on the parameters of decay-wave-pulse-type electroporator BTX ECM630 (conditions of 800 V, 1575 Ω, 50 μF lead to the pulse length of 10–14 ms for the successful transformations) [25,31]. Low voltage mode (5–500 V) was used for the remaining trials.

Therefore, the low voltage combined with multiple pulse numbers were considered to provide enough electric power to introduce exogenous DNA into the cell. Next, pulses at 2–4 with 300, 400, or 500 V were evaluated (Figure 1A). Voltage of 500 V with 2 pulses was the better condition for transformation and voltage of 300 or 400 V could reach the fair good efficiencies. However, if the pulse number was increased at the condition of 500 V, almost none transformants were obtained (Figure 1A). Excessive duration of electroporation was probably the reason to make cells death, leading the low transformation frequency. In addition, the above data implied that decreasing pulse length and increasing pulse number could be the alternative way to enhance the transformation frequency.

To maximize transformation frequency using BTX ECM830, different combinations of increasing pulse numbers (2, 4, 6, 8, 10) and decreasing pulse lengths (12, 10, 8, 6, 4, 2 ms) were evaluated at voltage of 500 V. From these results, pulse length of 4 ms with 6 pulses was the best combination for high yield (368 ± 102 transformants) (Figure 1B). For other conditions (8 pulses with 4 ms or 6 pulses with 6 ms), they could reach a fair good transformation efficiency. Thus, the considerable transformation efficiency could obtain under multiple combinations with square-wave-pulse-type electroporator, indicating more suitable for electroporation. Unexpected at pulse length of 2 ms, even increasing pulse number did not increase a lot for the transformation efficiency. The reason probably was that every single pulse length was too short to have enough energy for exogenous DNA delivery into the cells.

### Comparison DNA delivery method for 21gr wild-type strain

**Figure 2:**
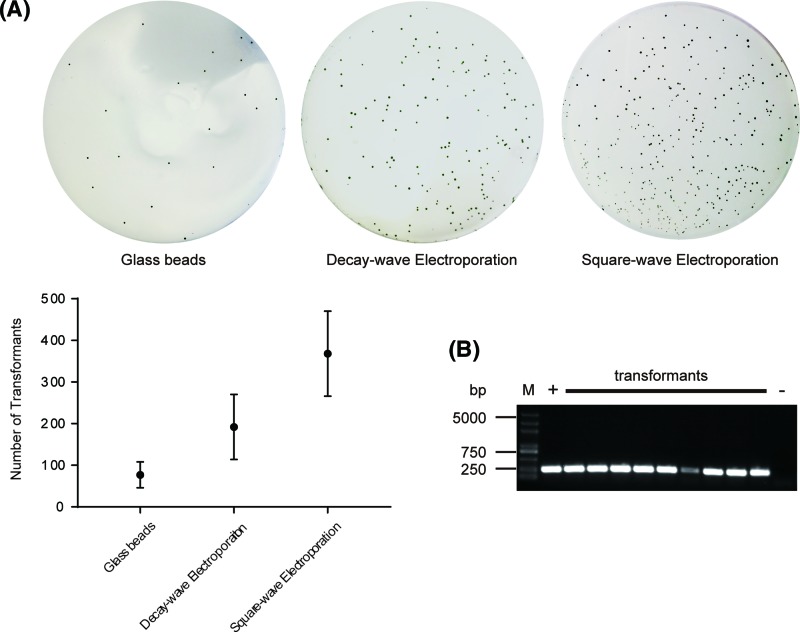
Transformation effects of *C. reinhardtii* strain 21gr (**A**) Colonies from glass beads method (top, left), decay-wave electroporation (top, middle), and square-wave electroporation (top, right) on one representative TAP agar plate containing 10 μg ml^−1^ paromomycin. The corresponding transformants numbers were plotted as mean ± SEM in three independent experiments (bottom). (**B**) Transformants confirmed by colony PCR. Genomic DNAs of nine transformants were randomly selected and confirmed by the expected band (264 bp) with primer F1 (5′-GATTCCCGTACCTCGTGTTG-3′) and R1 (5′-TCGTCCAGATCCTCCAAGTC-3′). M, Trans2K Plus DNA Marker (TransGen Biotech, China). +, pJMG plasmid (carrying *aphVIII* fragment) as positive control. −, wild-type 21gr genomic DNA as negative control.

To elucidate the superiority of the square-wave electroporation method the traditional DNA delivery method with glass beads and electroporation with decay-wave-pulse-type electroporator BTX ECM630 were also evaluated at their optimized conditions. The transformation efficiency at optimized conditions with square-wave electroporation method was 368 ± 102 cells, while the number of transformation with glass beads method was only 77 ± 31 cells (20.9% of efficiency of square-wave electroporation), and with the decay-wave method 192 ± 78 cells (52.2% of efficiency of square-wave electroporation) were obtained (Figure 2A). Thus, square-wave electroporation method is more applicable for transformation with *C. reinhardtii* cells for insertional mutant library construction.

### Optimized electroporation method for other wild-type strains

To determine whether this electroporation method was applicable to other wild-type cells with the cell wall, we applied this method to other cell-walled strain 6145C, CC-124 and CC-125 (Table 1). For strain 21gr, the transformation efficiency at conditions of 6 pulses (368 ± 102 cells) was better than that of 7 pulses (239 ± 31 cells). On the contrary, the transformation efficiency of 6145C at 6 pulses (256 ± 136 cells) was a little bit decreased compared with that of 7 pulses (320 ± 161 cells). However, the numbers of transformants obtained from these two conditions were basically at the same level. Different transformation efficiencies were acquired for the other two wild-type cells. For CC-124, the transformation efficiency at 7 pulses (624 ± 251 cells) was almost twice than that of 6 pulses (387 ± 151 cells). However, the transformation efficiency for CC-125 was almost the same low at both conditions (176 ± 80 cells at 6 pulses compared with 127 ± 76 cells at 7 pulses). In summary, the transformation efficiency for CC-125 was approximately 30% of that of CC-124, 50% of that of 21gr or 6145C, suggesting CC-125 was a difficult-to-transform strain. A similar result for CC-125 was obtained in the previous studies [24].

**Table 1:** Transformation efficiency of various *C. reinhardtii* strains

Strain	Electric conditions^a^ (voltage, pulse length, pulse number)	Number of transformants^b^
21gr (CC-1690)	500 V, 4 ms, 6 pulses	368 ± 102
	500 V, 4 ms, 7 pulses	239 ± 31
6145C (CC-1691)	500 V, 4 ms, 6 pulses	256 ± 136
	500 V, 4 ms, 7 pulses	320 ± 161
CC-124	500 V, 4 ms, 6 pulses	387 ± 151
	500 V, 4 ms, 7 pulses	624 ± 251
CC-125	500 V, 4 ms, 6 pulses	176 ± 80
	500 V, 4 ms, 7 pulses	127 ± 76

a 100 ng *aphVIII* DNA fragments and 5 × 10^7^ cells were used for each trial; pulse interval was 100 ms.

b Mean ± SEM (*n*=3).

In summary, the final optimized electroporation parameters applicable for the transformation of the four widely used wild-type strains were listed in Table 2. Electroporation of 5 × 10^7^ cells in the total volume of 250 μl cell suspension with 100 ng *aphVIII* fragments under conditions of 500 V with 4 ms pulse length, 100 ms pulse interval, and 6 pulse number resulted in an average of 3680 transformants per μg DNA for strain 21gr, 3200 transformants per μg DNA for strain 6145C, 6240 transformants per μg DNA for strain CC-124, and 1760 transformants per μg DNA for strain CC-125, which were higher than those in the previous electroporation technique.

**Table 2:** Optimal electroporation conditions for *C. reinhardtii* cells

**Sample conditions**	
Sample size	250 μl in 0.4 cm electroporation cuvette
Cell number	5 × 10^7^ cells per cuvette
Exogenous DNA	100 ng per cuvette (400 ng ml^−1^)
Transformation medium	TAP + 60 mM sorbitol
Electric conditions	
Voltage	500 V
Pulse length	4 ms
Pulse number	6–7 pulses
Pulse interval	100 ms

### Colony PCR for analysis of transformants

To determine exogenous *aphVIII* fragments were truly integrated into the genome of transformants, colony PCR for *Chlamydomonas* cells was applied [30]. After transformation for 21gr with *aphVIII* fragments, nine transformants on the TAP plate with paromomycin were randomly selected for colony PCR (Figure 2A). The 264 bp DNA fragment was amplified. The expected PCR products were obtained from all the nine transformants (Figure 2B), which demonstrated *aphVIII* fragment was truly integrated into the genome of transformants. The transformants with randomly insertional mutations were then screened for phenotypes of interest and mapped for the insertional sites.

## Discussion

To date, nuclear transformation technique for *C. reinhardtii* cells is urgently needed to develop for genetically understanding the mechanisms of biological processes or genetically engineering the cells for bioindustry*.* Especially for the forward genetic screen, selectable marker fragments should be as less as possible, as to make insertional sites as fewer as possible. Here, we reveal an optimized square-electric-pulse electroporation technique to introduce exogenous DNA into the genome of several wild-type strains of *C. reinhardtii*.

Transformation of *C. reinhardtii* cells with cell wall by square-wave pulse generator BTX ECM830 is never reported. To acquire the optimized conditions of square-wave pulse generator BTX ECM830, the published electroporation parameters of BTX ECM630 and NEPA21 were seriously considered. BTX ECM630 is an exponential decay-wave electroporation generator, while NEPA21 is a square decay-wave electroporation generator. Both are applicable for the transformations of *C. reinhardtii* cells. For electroporator ECM630, transformation efficiency with the optimized conditions was 2–3 × 10^3^ transformants per μg exogenous DNA fragments, while application of electroporator NEPA21 result in 0.4–3 × 10^3^ transformants per μg exogenous DNA fragments [24,25]. With the optimal electroporation procedure in BTX ECM830, the final yields of transformants in all three strains (1760, 3680, 6240 colonies per μg DNA for CC-125, 21gr, CC-124, respectively) were better than those with the previous electroporation method (404, 3400, 2930 colonies per μg DNA for CC-125, 21gr, CC-124, respectively) [24]. Various electroporation efficiencies may reflect diverse characteristics of different strains in the cell wall, cytoplasmic membrane, cytosol, and even nucleus. The wild-type CC-125 may be a rigid strain for electroporation based on previous and present studies, which should be seriously considered when using this wild-type strain for electroporation. Thus, the optimal conditions for electroporation of *C. reinhardtii* are also the strain-dependent conditions. Meanwhile, the number of transformants obtained by glass beads method was low and varied greatly, depending on the activity of the autolysin. Thus, the glass beads method is not suitable for mutant library construction. The controllable pulse lengths with square-wave pulse generator make it the better choice for building mutant library than that with decay-wave generator.

In addition, fewer exogenous DNA fragments were applied in this procedure that could introduce fewer copies or even one copy of exogenous DNA into the genome. The estimated copy numbers of the transformants were 1.8 and 3.0 copies with the transformations of 2.5 and 10 μg ml^−1^ DNA [23]. Additionally, 400 ng exogenous DNA fragments in the volume of 40 μl cell suspension (10 μg ml^−1^) were applied in the method of NEPA21 with 4 × 10^6^ cells [24]. However, only 400 ng ml^−1^ DNA employed in this procedure (100 ng DNA with 5 × 10^7^ cells in the volume of 250 μl cell suspension) would introduce less than 1.8 copies into the genome. With this advantage, it is more applicable to explore the insertional site for genetic studies and further analysis.

High concentration of DNA truly increases the transformation efficiency [23,24], which is more suitable for the transgene expression system. For generating tagged protein expressed cell line or rescue of mutants, multiple copies of exogenous DNA integrated into the genome may be not a big problem. In our experiences, the amount of plasmids expressing tagged protein or cosmids should be increased for electroporation. It is probably the size-dependent manner for DNA entry into the cell. To easily gain transgene expressed cell line by electroporation, the construct carrying both the tagged transgene and the selection marker gene for uni-transformation instead of co-transformation is the best way [4,11].

Hence, square-wave electroporation technique applied here is currently a rapid and easy way to introduce exogenous DNA into the genome of the *C. reinhardtii* cell with high efficiency, more applicable for mutants screening.
